# The HOPE cohort: cohort profile and evaluation of selection bias

**DOI:** 10.1007/s10654-024-01150-4

**Published:** 2024-08-19

**Authors:** Mette-Marie Zacher Kjeldsen, Merete Lund Mægbæk, Xiaoqin Liu, Malene Galle Madsen, Mette Bliddal, Sofie Egsgaard, Kathrine Bang Madsen, Trine Munk-Olsen

**Affiliations:** 1 NCRR-National Centre for Register-Based Research, Aarhus University, Aarhus, Denmark; 2 Department of Public Health, Aarhus University, Aarhus, Denmark; 3 CIRRAU – Centre for Integrated Register-Based Research, Aarhus University, Aarhus, Denmark; 4 Research Unit OPEN, Department of Clinical Research, University of Southern Denmark, Odense, Denmark; 5 Department of Clinical Research, University of Southern Denmark, Odense, Denmark; 6 Child and Adolescent Psychiatric Unit, Region of Southern Denmark, Odense, Denmark

**Keywords:** Postpartum depression, Edinburgh postnatal depression scale, Cohort profile, Selection

## Abstract

The HOPE cohort is a Danish nationwide cohort with ongoing follow-up, holding information on postpartum depression (PPD) symptoms and diagnoses on 170,218 childbirths (142,795 unique mothers). These data have been linked with extensive register data on health and socioeconomic information on the mothers, their partners, parents, and children. This cohort profile aimed to provide an overview of the data collection and content, describe characteristics, and evaluate potential selection bias. PPD screenings, using the Edinburgh Postnatal Depression Scale, were collected from 67 of the 98 Danish municipalities, covering the period January 2015 to December 2021. This data was linked with register data on PPD diagnoses (identified through medication prescriptions and hospital contacts) as well as background information. Cohort characteristics were compared to the source population, defined as all childbirths by women residing in Denmark during the same period (452,207 childbirths). Potential selection bias was evaluated by comparing odds ratios of five well-established associations between the cohort and the source population. The HOPE cohort holds information on 170,218 childbirths (38% of the source population) involving 142,795 unique mothers. The HOPE cohort only differed slightly from the source population on most characteristics examined, but larger differences were observed on specific characteristics with an underrepresentation of the youngest and oldest age groups, women with more than three children or twins/triplets, and women born outside Denmark. Similar associations were identified across the two populations within the five well-established associations. There was no indication of selection bias on the five examined associations, and the HOPE cohort is representative of the source population on important perinatal characteristics.

**Supplementary Information:**

The online version contains supplementary material available at 10.1007/s10654-024-01150-4.

## Introduction

The HOPE cohort is a prospective cohort established in 2022, containing nationwide data on postpartum depression (PPD) symptoms (screenings) and recorded diagnoses (medication prescriptions and hospital contacts) for the period 2015 to 2021 in Denmark. The cohort is linked with extensive and continuously updated national register data, including information on important PPD risk factors. This linkage enables follow-up of the entire cohort, as well as partners, children, and extended relatives. To the best of our knowledge, this is the largest PPD cohort to date, encompassing information on the entire spectrum of recorded episodes of maternal PPD, ranging from mild to severe cases. The cohort covers 170,218 childbirths of 142,795 unique mothers. It serves as a source for ongoing research within the field of PPD, designed to lay the foundation for a deeper understanding of the interrelated risk factors, treatment patterns, and short- and long-term consequences of PPD in the mother, the father, and their children. This new knowledge is crucial for clinical practice and healthcare planning to develop more targeted prevention and treatment strategies.

In the Western world, PPD affects 10–15% of new mothers, making it the most prevalent psychiatric disorder related to childbirth [1–4]. Severe short- and long-term consequences have been observed for both the new mother and her child [2]. Feasible and effective treatment for PPD exists [5–7]; however, to initiate timely identification of high-risk women, knowledge about the causes of PPD is needed. For this purpose, numerous systematic reviews have been published on single and multiple risk factors, and three comprehensive umbrella reviews summarizing knowledge from these systematic reviews have been published [8–10]. Some of the most significant risk factors identified include personal and family history of psychiatric disorders, as well as different pregnancy- and birth-related complications [8–11]. However, it is believed that the aetiology of PPD is explained by a combination of several risk factors, and recently, risk prediction models have emerged as a first step toward a better understanding of the interrelated causes of PPD [12].

In general, research on PPD has often relied on measures of symptoms, such as using the screening tool Edinburgh Postnatal Depression Scale (EPDS) for case identification. The EPDS is developed specifically for the postpartum period and is validated in several languages, including Danish, making it widely used around the world [13–15]. In the Nordic countries, much PPD research has been based on nationwide registers as they contain information on the entire population, covering various aspects of life, including health, disease, and social conditions [16–18]. PPD research conducted using Nordic registers has primarily focused on medication use and hospital contacts for case identification, identifying the more severe psychiatric disorders requiring hospital contacts or psychotropic medication treatment. However, this limits generalization of findings to severe cases only, as information on milder cases and subclinical symptoms is not recorded in any registers [19].

The Danish Health Authority recommends PPD screening in all municipalities during healthcare nurse visits to the family [20]. Thus, screening is highly prevalent in Denmark and is most often conducted around eight weeks postpartum by healthcare nurses using the EPDS [20, 21]. Contrary to the population registers, PPD screenings have not been available for research purposes. Based on efforts from our group, a large sample of these EPDS screenings has now been collected and linked with register data on PPD diagnoses, forming the HOPE cohort.

Screening practices vary significantly in Denmark, exhibiting discrepancies related to who (target group), how (screening tools), and when (assessment time point) to screen [20, 21]. If healthcare nurses are more inclined to screen mothers whom they think are at a higher risk of developing PPD and on suspicion of current PPD, selection bias could arise. This unknown selection into the cohort is of potential concern, making an examination of selection bias in the HOPE cohort in relation to the source population essential. Hence, the purpose of this paper is to provide an overview of data collection and content in the cohort, describe characteristics of cohort members, and evaluate selection bias in relation to the source population. Additionally, potential for future use of the cohort will be outlined.

## Methods

### Eligibility and inclusion in the HOPE cohort

Healthcare nurse service is universal in Denmark and free of charge, with several visits during the initial eight months postpartum [21]. Screening for PPD is frequently completed at the visit eight weeks postpartum, most often using the EPDS [21]. Permission from the Danish Patient Safety Authority was obtained to receive these questionnaires for the period January 1, 2015, to December 31, 2021, and from the Danish Data Protection Agency to establish a research project linked with register data hosted by Statistics Denmark. Permission from the Danish Patient Safety Authority was granted for the 88 out of 98 municipalities who use a specific software system, NOVAX, for storage of their healthcare nurse data, on the condition that the municipalities gave consent to provide data to the cohort.

**Fig. 1:**
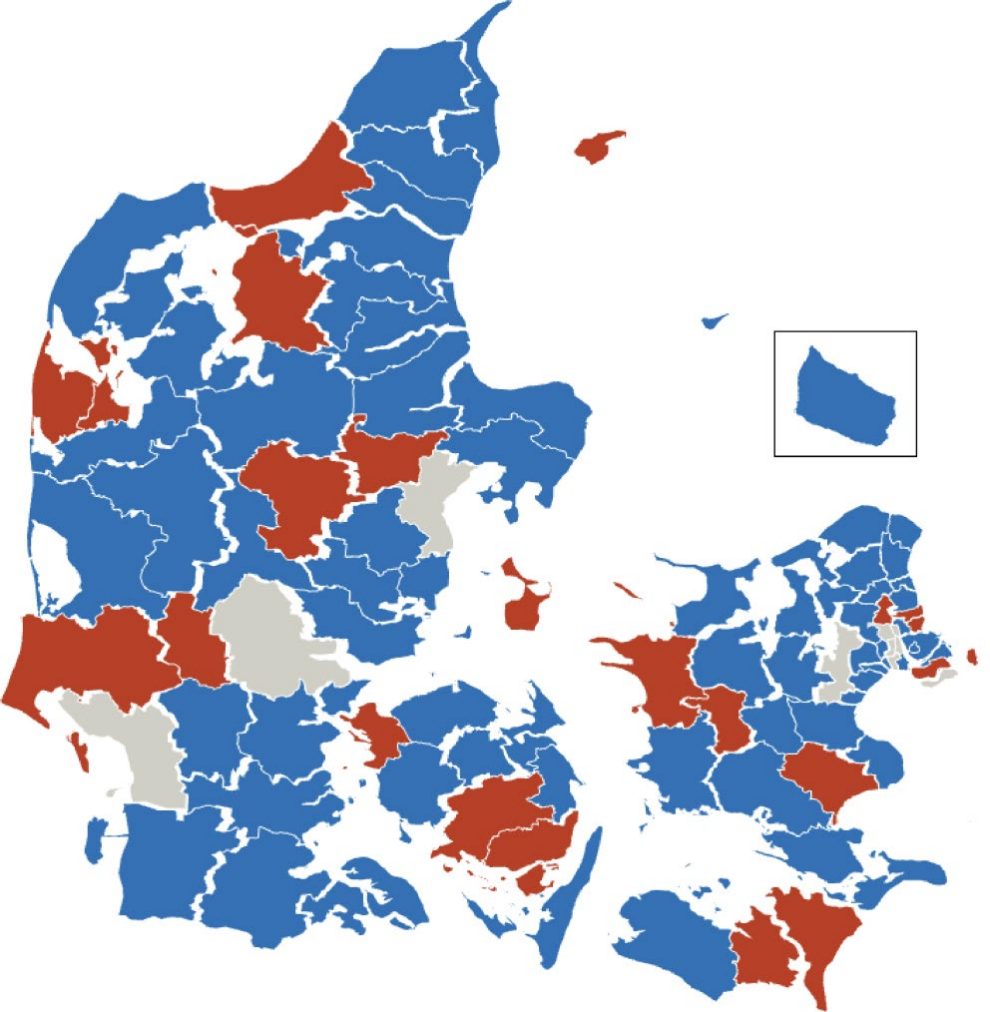
Map of the participating, non-participating, and non-eligible Danish municipalities. Blue = participating municipalities, red = non-participating municipalities, grey = non-eligible municipalities

The leading healthcare nurses in the 88 eligible Danish municipalities were contacted by email by the corresponding author, MMZK, followed by one or several phone calls from October 2021 through March 2022, to obtain consent to access their EPDS data. Of the 88 municipalities, 67 (76%) gave consent to provide data for the cohort (Fig. 1). Of the 21 municipalities not participating, 10 declined, 9 did not respond to the request, and 2 did not store EPDS data in NOVAX, after all.

**Fig. 2:**
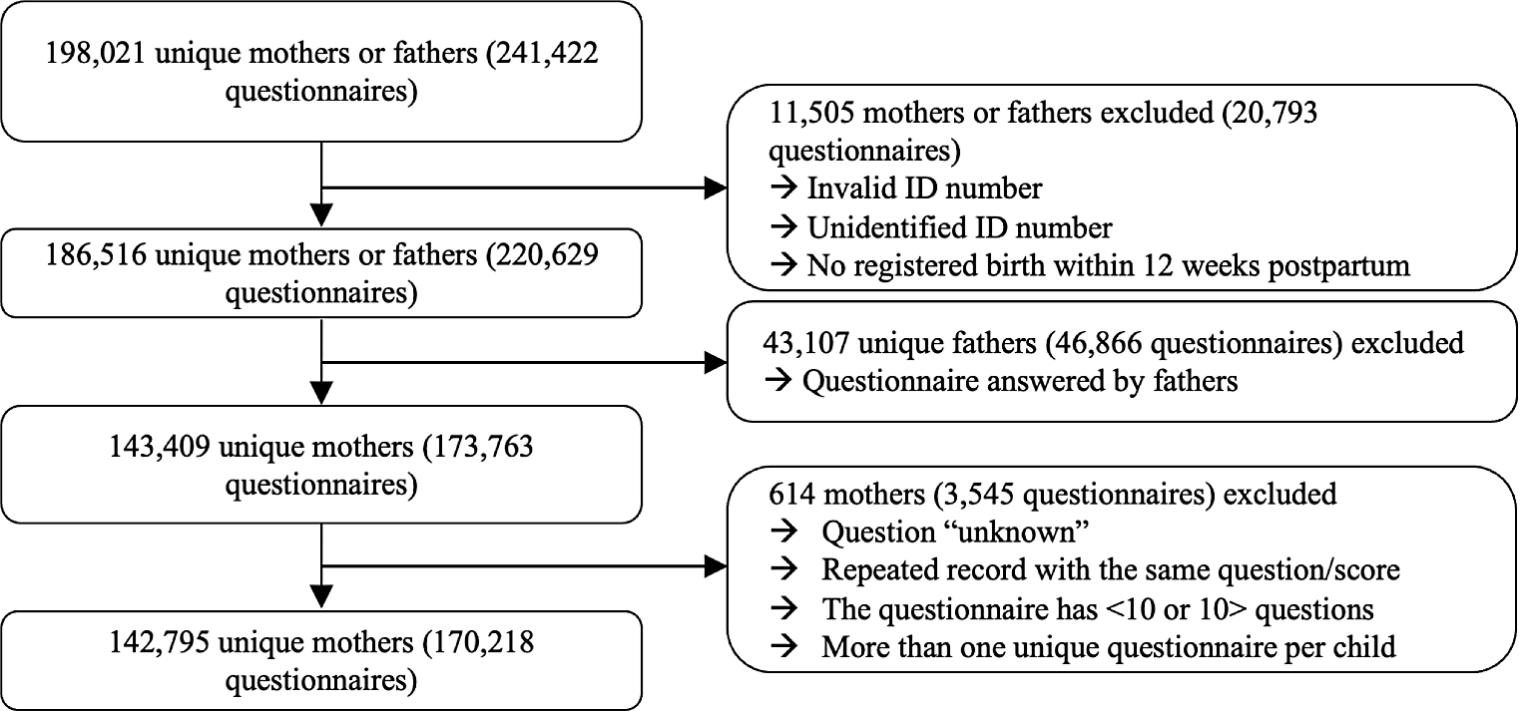
Flowchart of the HOPE cohort

A dataset comprising EPDS data from the 67 included municipalities was transferred from NOVAX to a secure server at Aarhus University, representing 198,021 unique mothers or fathers (241,422 questionnaires). All Danish residents are assigned a unique 10-digit Danish Civil Registration number at birth or immigration, and using the Danish Civil Registration System, data were merged with the personal ID numbers on Statistics Denmark [22]. To account for variation in assessment time points and potential delays with electronic registration of the EPDS, we restricted to questionnaires conducted within 12 weeks postpartum. Consequently, we excluded 11,505 mothers or fathers (20,793 questionnaires) due to either an unidentified or invalid ID number or not having a registered birth within 12 weeks of filling in the questionnaire. Further, 43,107 unique persons (46,866 questionnaires) were excluded for the present study, as the respondent was registered as the father of the child. This subset of data will be investigated in detail in a separate project. Of the remaining 143,409 unique mothers (173,763 questionnaires), a further 614 (3,545 questionnaires) were excluded as we restricted to questionnaires being complete, containing exactly 10 questions, and limited to only one unique questionnaire within 12 weeks after childbirth. Consequently, our final sample comprised 142,795 unique mothers of 170,218 unique childbirths, and thus 170,218 unique EPDS questionnaires, all of whom were included in the HOPE cohort (Fig. 2).

### Data in the HOPE cohort

Using the unique ID numbers for each participant in the Danish Civil Registration System, each mother with an EPDS screening included in the HOPE cohort was linked with information from several registers through Statistics Denmark [22]. An overview of these registers and accompanying information is presented in Table 1.

**Table 1:** Overview of data in the HOPE cohort

Domain (register/questionnaire)	Content
EPDS questionnaires	The EPDS consists of 10 items, each scored between 0 and 3, totalling a score range of 0–30. Cut off on 11 or above indicating PPD according to the Danish validation of the scale [15]. The HOPE cohort holds EPDS screenings conducted in the period January 1, 2015, to December 31, 2021 (restricted to screenings conducted within 12 weeks postpartum): Maternal EPDS screenings: 170,218 childbirths from 142,795 unique mothers. Paternal EPDS screenings: 46,563 childbirths from 43,059 unique fathers. Parental dyads (mothers and fathers): 45,738 childbirths.
The National Patient Register [23]	Inpatient admission to hospital with somatic disorders from 1977 onwards. From 1994 registration of outpatient somatic disorders started (complete from 1995). From 1995 onwards, inpatient, emergency, and outpatient contacts to psychiatric facilities, including psychiatric diagnoses.
The Psychiatric Central Research Register [24]	Admission to specialized psychiatric treatment facilities including psychiatric diagnosis for the period 1969 onwards. From 1995 outpatient treatment and emergency room contacts with psychiatric diagnosis was added.
The National Prescription Register [25]	Redeemed prescriptions for the overall ATC categories A, J, N, and R from 1995 onwards.
The Medical Birth Register [26]	Linkage between mother and child, pregnancy, and birth-related contacts to hospital from 1973 onwards.
The Register on Causes of Death [27]	Information on causes of death from 1973 onwards.
The In Vitro Fertilisation (IVF) Register [28]	IVF treatments from 1994 onwards.
The Danish National Child Health Database [29]	Breastfeeding (from 2012), continuous weight and height measures of the child (from 1998), and child exposure to passive smoking (from 2009).
The Danish Central Crime Register	Information on convicted crimes from 1980 onwards.
The Health Insurance Register	Contact to family physicians, psychologists etc. from 1990 onwards.
Statistics Denmark’s registers on demography and socioeconomic status [30, 31]	Demography, family, cohabitation status, household information, employment, education, income.
Out-of-home placement of children	Children placed in out-of-home care.
Statistics Denmark’s registers on preventive measures for vulnerable families, children, and young people [32]	Preventive measures for vulnerable individuals from 1994 onwards.

### Follow-up

Using the personal ID number, continuous future updates of the register data are planned, with the potential of adding supplemental and prolonged follow-up information.

### Data access

Data are stored on a secure server at Statistics Denmark, and restrictions apply to availability of this dataset. Access can potentially be granted through collaboration with the HOPE research group and will be considered on a case-by-case evaluation by members of a steering group. Future studies conducted on the HOPE cohort must focus on perinatally related topics due to the permission data relies on.

### Characteristics of the HOPE cohort

We compared characteristics in the HOPE cohort to the source population. Since inclusion into the HOPE cohort was restricted to women with an EPDS screening conducted within 12 weeks postpartum from January 1, 2015, to December 31, 2021, the cohort members gave birth from October 2014 through December 2021. Accordingly, the source population was defined as all births by women residing in Denmark during the same period.

Variables included in the comparison were obtained, defined, and categorized as follows: Information on PPD diagnosis was retrieved from the National Patient Registry and the National Prescription Register, defined as either an in- or outpatient contact to a specialized psychiatric treatment facility with a depressive episode (International Classification of Diseases, version 10 (ICD-10) codes: F32-33) or a redeemed antidepressant prescription (Anatomical Therapeutic Chemical (ATC): N06A) within 6 months postpartum, to account for delay in PPD treatment, and categorized as *yes* or *no* [23, 25]. From the Civil Registration System, we obtained information on maternal age at delivery categorized as *< 20 years*, *20–24 years*, *25–29 years*, *30–34 years*, *35–39 years*, *40–44 years*, and *≥ 45 years*, calendar year of delivery categorized separately for each year from 2014 to 2021, and country of origin categorised as *Denmark* or *foreign/unknown* [22]. Cohabitation status was derived from the Population Statistics Register and coded as *cohabitating* or *not cohabitating*. Information on the highest attained maternal education was obtained from Statistics Denmark’s Register of Education and categorized as *mandatory*, *short*, *medium*, and *high* according to the International Classification of Education [30, 33]. The Medical Birth Register informed on parity categorized as *1*, *2*, *3*, and *≥ 4* children, and singleton births categorized as *yes* or *no* [26]. The Obstetric Comorbidity Index is an index for assessing the risk of morbidity or mortality in obstetrics and has been validated in a Danish context [34, 35]. Information needed to calculate the Obstetric Comorbidity Index was obtained from the Medical Birth Register and the National Patient Register [23, 26]. The index was defined according to Bateman and categorized according to previous work as *0*, *1*, *2*, or *≥ 3* [34, 36, 37]. Information on maternal hospital depression diagnosis within one year prior to delivery was obtained from the National Patient Registry and defined as a depressive episode (ICD-code: F32-33) within one year prior to delivery and categorized as *yes* or *no* [23]. Information on maternal antidepressant use within one year prior to delivery was obtained from the National Prescription Register and defined as a redeemed antidepressant medication prescription within one year prior to delivery (ATC-code: N06A) and categorized as *yes* or *no* [25]. Personal history of psychiatric disorders was obtained from the National Patient Registry and defined as any in- or outpatient visits to psychiatric facilities with psychiatric diagnosis (F00-F99) from 1995 to delivery in the index mother [23]. Information on family history of psychiatric disorders was obtained from the National Patient Registry and defined as any in- or outpatient visits to psychiatric facilities with psychiatric diagnosis (F00-F99) from 1995 to delivery in the index mothers’ parents [23]. Information on gestational diabetes mellitus was obtained from the Medical Birth Register and defined according to ICD-10 (ICD-10 code: O24) and categorized as *yes* or *no* [26]. Information on macrosomia was obtained from the Medical Birth Register and defined as a birth weight of > 4.0 kg. and categorized as *yes* or *no* [26, 38]. Information on smoking during pregnancy was obtained from the Medical Birth Register and categorized as *yes* or *no* [26]. Information on babies born small for gestational age (SGA) was obtained from the Medical Birth Register and defined as a birth weight below the 10th percentile restricted to children with a birth weight > 300 g and < 6400 g and born within gestational week 23–45, and categorized as *yes* or *no* [26, 39]. Information on maternal pre-pregnancy body mass index (BMI) was obtained from the Medical Birth Register and calculated as weight in kilograms divided by the square of height in meters and defined according to the World Health Organization’s (WHO) pre-defined BMI groups: *<18.5*, *18.5–24.9*, *25-29.9*, *≥ 30* restricted to women with a BMI > 12 and < 50 [26, 40]. Information on acute caesarean section (C-section) was obtained from the National Patient Registry and the Medical Birth Register and defined according to ICD-10 (ICD-10 codes: DO821, DO843, DO829, DO842 and procedure codes: KMCA10A, KMCA10E) and categorized as *yes* or *no* [23, 26].

### Evaluating potential selection bias

**Fig. 3:**
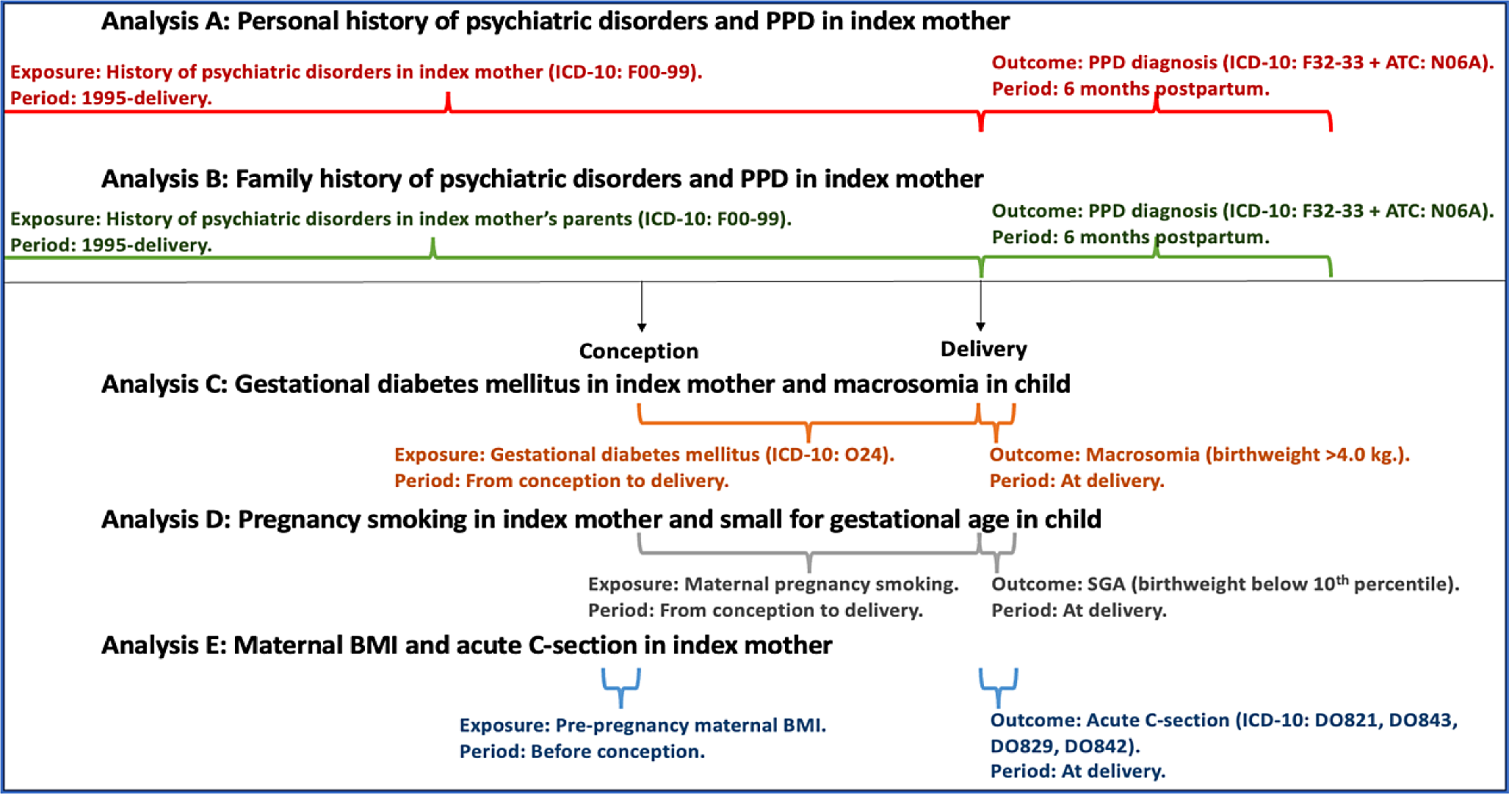
Details on definitions of exposures and outcomes in analysis **A-E**

A potential limitation for almost all observational cohorts is selection bias. The extensive Danish registries provide an opportunity to examine potential selection bias in the HOPE cohort, as information on the source population is available. We examined if inclusion into the HOPE cohort influenced five well-established perinatal exposure-outcome associations when compared to the source population:

A) Personal history of psychiatric disorders and risk of PPD identified through diagnosis [4, 41, 42],
B) Family history of psychiatric disorders and risk of PPD identified through diagnosis [43],
C) Gestational diabetes mellitus and risk of macrosomia [44],
D) Smoking during pregnancy and risk of SGA [45], and.
E) Maternal pre-pregnancy BMI and risk of acute C-section [46].
F) Variables for the analyses were obtained, defined, and categorized according to variables used for characteristics. Only exception was age since it was used as a continuous variable in the evaluation of selection bias. See Fig. 3 for details on each of the five exposure-outcome associations.

We employed three separate analysis populations for the five analyses: one population for analysis A (analysis population A), one population for analysis B (analysis population B), and a third population for analyses C, D, and E (analysis population CDE).

Analysis population A: Childbirths in the HOPE cohort commenced from October 2014 onwards. Inclusion ended 6 months prior to the end of follow-up to allow for 6 months follow-up of the outcome (PPD). Hence, inclusion ended on June 30, 2021, and follow-up concluded on December 31, 2021. The source population was accordingly defined as all births in Denmark from October 2014 to July 2021. To prevent misclassification of the outcome (PPD), we applied a washout period, excluding all index mothers with a depressive episode (ICD-10: F32-33 or ATC-code: N06A) one year prior to delivery.

Analysis population B: Same population as in analysis population A, but with an extra exclusion of index mothers without information on the index mothers’ mother or father, since family history of psychiatric disorders was of primary interest.

Analysis population CDE: Inclusion was from October 2014 to December 2021 for both the cohort members and the source population. No washout period was applied.

### Statistical analysis

#### Characteristics of the HOPE cohort

We calculated frequencies of selected characteristics in the HOPE cohort and the source population. Prevalence ratios (PR) were then computed by dividing the frequency in the HOPE cohort by the corresponding frequency in the source population, accompanied by 95% confidence intervals (CI). PRs were used to evaluate direction and magnitude of potential selection bias: underrepresentation (PR < 1) or overrepresentation (PR > 1) of specific characteristics among cohort members compared to the source population. Estimation of 95% CIs for PRs was done by calculating standard errors, since the two populations were not independent [47].

### Evaluation of selection bias

To estimate each of the five exposure-outcome associations, we utilized logistic regressions applying Generalized Estimating Equations (GEE) methods to account for instances where women were included more than once in the HOPE cohort (e.g., giving birth to more than one child in the period) [48]. Analysis population CDE was further restricted to singletons, as outcomes in analyses C and D focused on birth weight (macrosomia and SGA, respectively). Complete case analysis was conducted within each analysis population. Results were presented as crude and adjusted odds ratios (ORs) with 95% CIs in both the HOPE cohort and the source population. Adjusted analyses included maternal age, parity, and education. Maternal age was included in the model as a continuous variable modelled by a restricted cubic spline, as age was not linear on the log-odds scale. Relative ORs (ROR) were estimated as the ratio between the adjusted OR in the HOPE cohort and the source population. RORs were likewise estimated to evaluate potential selection bias: underestimation (ROR < 1) or overestimation (ROR > 1) of the specific association among cohort members. The 95% CIs for ORs and RORs were calculated following the same procedure as for the calculation of CIs for the PRs [47].

### Sensitivity analyses

Two sensitivity analyses were conducted: one restricting analysis population AB to singletons to account for differences between singletons and twins/triplets, and another presenting characteristic of cohort members and the source population restricted to Danish-born women to account for missing information in the Danish registers among non-Danish born women.

## Results

### Characteristics of the study population

Distribution of selected characteristics in the two groups, the HOPE cohort and the source population, along with PRs and 95% CIs, is presented in Table 2. Of the source population, comprising 452,207 childbirths, 170,218 were included in the HOPE cohort (38%).

Minor differences were observed between the HOPE cohort and the source population on the following characteristics: Slightly more women in the HOPE cohort had a PPD diagnosis (PR, 1.06 [95% CI, 1.06;1.06]), used antidepressant medication one year prior to delivery (PR, 1.03 [95% CI, 1.02;1.03]), were less likely to have received a hospital depression diagnosis one year prior to delivery (PR, 0.96 [95% CI, 0.96;0.97]), had more often a personal (PR, 1.07 [95% CI, 1.07;1.08]) or family history of psychiatric disorders (PR, 1.07 [95% CI, 1.07;1.08]), were more likely to have a cohabitant partner (PR 1.01 [95% CI, 1.01;1.01]), were more likely to have higher educational levels, were less likely to smoke during pregnancy (PR, 0.99 [95% CI, 0.99;1.00]), had more healthy BMI, and had fewer obstetric comorbidities (Obstetric Comorbidity Index ≥ 3 PR, 0.96 [95% CI, 0.95;0.96], GDM PR, 0.96 [95% CI, 0.96;0.96], macrosomia PR, 1.06 [95% CI, 1.06;1.07], SGA PR, 1.03 [95% CI, 1.03;1.04]).

Larger differences between the two populations were observed on specific characteristics. The HOPE cohort was underrepresented in the youngest and oldest age groups (PR, 0.69 [95% CI, 0.69;0.70] and 0.75 [95% CI, 0.75;0.76]), among women born outside Denmark (PR, 0.58 [95% CI, 0.58;0.58]), among those giving birth to twins/triplets (PR, 0.80 [95% CI, 0.80;0.80]), and among women with 4 + children (PR, 0.64 [95% CI, 0.64;0.64]). However, it is important to notice that despite large relative differences between the HOPE cohort and the source population on these characteristics, absolute differences were in fact not large in relation to the youngest (prevalence 0.5% vs. 0.8%) and oldest (prevalence 0.2% vs. 0.3%) age groups, women with 4 + children (prevalence 2.3% vs. 3.5%), and women giving birth to twins/triplets (prevalence 1.2% vs. 1.5%). When looking at women of non-Danish origin, the differences between the HOPE cohort and the source population were in fact large in both relative (PR, 0.58 [95% CI, 0.58;0.58]) and absolute measures (prevalence 13.5% vs. 23.3%). Furthermore, variables with missing information regarding education status, parity, singleton birth, family history of psychiatric disorders, macrosomia, smoking during pregnancy, SGA, and maternal pre-pregnancy BMI were much more pronounced in the source population than in the HOPE cohort.

Comparisons between the two populations for additional pregnancy- and birth-related complications, including preterm birth, postpartum haemorrhage, gestational hypertension, preeclampsia and/or eclampsia, previous abortion, and hyperemesis gravidarum, revealed a slight overrepresentation in the HOPE cohort of women with these complications, except women with preterm born babies who were underrepresented in the HOPE cohort (Supplement Table S1).

**Table 2:** Characteristics of the HOPE cohort and the source population with prevalence ratios and 95% confidence intervals

Variable	HOPE cohort (*n* = 170,218) *N* (%)	Source population (*n* = 452,207) *N* (%)	PR (95% CI)
PPD diagnosis			
Yes	5,339 (3.1)	13,367 (3.0)	1.06 (1.06–1.06)
No	164,879 (96.9)	438,840 (97.0)	1.00 (1.00–1.00)
Maternal age at delivery			
15–19 years	909 (0.5)	3,487 (0.8)	0.69 (0.69–0.70)
20–24 years	15,982 (9.4)	43,977 (9.7)	0.97 (0.96–0.97)
25–29 years	59,715 (35.1)	151,526 (33.5)	1.05 (1.04–1.05)
30–34 years	60,250 (35.4)	159,670 (35.3)	1.00 (1.00-1.01)
35–39 years	27,232 (16.0)	75,604 (16.7)	0.96 (0.95–0.96)
40–44 years	5,814 (3.4)	16,830 (3.7)	0.92 (0.91–0.92)
≥45 years	316 (0.2)	1,113 (0.3)	0.75 (0.75–0.76)
Calendar year of delivery			
2014	3,249 (1.9)	12,696 (2.8)	0.68 (0.68–0.68)
2015	21,049 (12.4)	61,774 (13.7)	0.91 (0.90–0.91)
2016	24,205 (14.2)	64,464 (14.3)	1.00 (0.99-1.00)
2017	25,818 (15.2)	63,465 (14.0)	1.08 (1.08–1.08)
2018	27,298 (16.0)	62,971 (13.9)	1.15 (1.15–1.16)
2019	27,500 (16.1)	62,280 (13.8)	1.17 (1.17–1.18)
2020	21,037 (12.4)	61,469 (13.6)	0.91 (0.91–0.91)
2021	20,062 (11.8)	63,088 (13.9)	0.84 (0.84–0.85)
Country of origin			
Denmark	147,235 (86.5)	346,788 (76.7)	1.13 (1.13–1.13)
Foreign/unknown	22,983 (13.5)	105,419 (23.3)	0.58 (0.58–0.58)
Cohabitation status			
Cohabitating	146,854 (86.3)	386,747 (85.5)	1.01 (1.01–1.01)
Not cohabitating	23,364 (13.7)	65,460 (14.5)	0.95 (0.94–0.95)
Education			
Mandatory	16,354 (9.6)	53,363 (11.8)	0.81 (0.81–0.82)
Short	50,974 (29.9)	127,582 (28.2)	1.06 (1.06–1.06)
Medium	8,842 (5.2)	22,569 (5.0)	1.04 (1.04–1.04)
High	93,423 (54.9)	231,882 (51.3)	1.07 (1.07–1.07)
Missing	625 (0.4)	16,811 (3.7)	0.10 (0.09–0.10)
Parity			
1	90,195 (53.0)	201,042 (44.5)	1.19 (1.19–1.19)
2	58,234 (34.2)	157,431 (34.8)	0.98 (0.98–0.99)
3	16,623 (9.8)	52,039 (11.5)	0.85 (0.85–0.85)
≥4	3,856 (2.3)	16,023 (3.5)	0.64 (0.64–0.64)
Missing	1,310 (0.7)	25,672 (5.7)	0.14 (0.13–0.14)
Singleton			
Yes	167,910 (98.6)	425,681 (94.1)	1.05 (1.05–1.05)
No	2,045 (1.2)	6,797 (1.5)	0.80 (0.80–0.80)
Missing	263 (0.2)	19,729 (4.4)	0.04 (0.03–0.04)
Obstetric Comorbidity Index			
0	114,279 (67.2)	304,361 (67.3)	1.00 (1.00–1.00)
1	35,961 (21.1)	94,095 (20.8)	1.02 (1.01–1.02)
2	11,789 (6.9)	31,029 (6.9)	1.01 (1.01–1.01)
≥3	8,189 (4.8)	22,722 (5.0)	0.96 (0.95–0.96)
Hospital depression diagnosis 1 year prior to delivery			
Yes	802 (0.5)	2,212 (0.5)	0.96 (0.96–0.97)
No	169,416 (99.5)	449,995 (99.5)	1.00 (1.00–1.00)
Antidepressant use 1 year prior to delivery			
Yes	5,870 (3.5)	15,179 (3.4)	1.03 (1.02–1.03)
No	164,348 (96.5)	437,028 (96.6)	1.00 (1.00–1.00)
Personal history of psychiatric disorders			
Yes	23,479 (13.8)	58,065 (12.8)	1.07 (1.07–1.08)
No	146,739 (86.2)	394,142 (87.2)	0.99 (0.99–0.99)
Family history of psychiatric disorders			
Yes	26,537 (15.6)	65,695 (14.5)	1.07 (1.07–1.08)
No	130,016 (76.4)	308,091 (68.1)	1.12 (1.12–1.12)
Missing	13,665 (8.0)	78,421 (17.4)	0.46 (0.46–0.47)
Gestational diabetes mellitus			
Yes	8,324 (4.9)	23.022 (5.1)	0.96 (0.96–0.96)
No	161,894 (95.1)	429,185 (94.9)	1.00 (1.00–1.00)
Macrosomia			
Yes	29,012 (17.0)	72,539 (16.0)	1.06 (1.06–1.07)
No	137,708 (80.9)	348,634 (77.1)	1.05 (1.05–1.05)
Missing	3,498 (2.1)	31,034 (6.9)	0.30 (0.30–0.30)
Smoking during pregnancy			
Yes	9,622 (5.7)	25,775 (5.7)	0.99 (0.99-1.00)
No	154,924 (91.0)	391,690 (86.6)	1.05 (1.05–1.05)
Missing	5,672 (3.3)	34,742 (7.7)	0.43 (0.43–0.44)
SGA			
Yes	16,307 (9.6)	41,899 (9.2)	1.03 (1.03–1.04)
No	150,276 (88.3)	378,845 (83.8)	1.05 (1.05–1.05)
Missing	3,635 (2.1)	31,463 (7.0)	0.31 (0.30–0.31)
Maternal pre-pregnancy BMI			
12-18.4	6,290 (3.7)	17,223 (3.8)	0.97 (0.97–0.97)
18.5–24.9	100,011 (58.8)	251,386 (55.6)	1.06 (1.05–1.06)
25-29.9	36,566 (21.5)	93,690 (20.7)	1.04 (1.03–1.04)
≥30–50	23,190 (13.6)	58,173 (12.9)	1.06 (1.06–1.06)
<12 or > 50	1,367 (0.8)	4,221 (0.9)	0.86 (0.86–0.86)
Missing	2,794 (1.6)	27,514 (6.1)	0.27 (0.27–0.27)
Acute C-section			
Yes	24,723 (14.5)	65,462 (14.5)	1.00 (1.00-1.01)
No	145,495 (85.5)	386,745 (85.5)	1.00 (1.00–1.00)

*Abbreviations* Postpartum depression (PPD), babies born small for gestational age (SGA), body mass index (BMI), caesarean section (C-section), prevalence ratio (PR), confidence interval (CI)

The median EPDS score with interquartile range (IQR) for each of the included years was 4 (IQR in the years 2015–2021: 2.00–7.00, IQR in the year 2014: 2.00–6.00). Among the 170,218 cohort members, 7.8% exhibited PPD symptoms according to the Danish EPDS validation [15]. Additionally, the average maternal age at first childbirth was 28 years in both populations.

### Evaluating selection bias in the HOPE cohort on selected exposure-outcome associations

Crude and adjusted ORs and RORs for the five perinatal exposure-outcome associations are presented in Table 3. For analyses A (personal history of psychiatric disorders and risk of PPD) and B (family history of psychiatric disorders and risk of PPD), the analysis population consisted of 155,970 and 143,360 childbirths, respectively (complete case analysis *n* = 154,173 and *n* = 142,064) after applying a restricted inclusion period and a washout period. For analyses C (gestational diabetes mellitus and risk of macrosomia), D (maternal pregnancy smoking and risk of SGA), and E (maternal pre-pregnancy BMI and risk of acute C-section), the analysis population consisted of all 170,218 childbirths (complete case analysis *n* = 159,184) in the HOPE cohort. The adjusted RORs were 0.94 (95% CI: 0.85–1.02) for the association between personal history of psychiatric disorders and risk of PPD diagnosis, 1.08 (95% CI: 0.98–1.18) for the association between family history of psychiatric disorders and risk of PPD, 0.98 (95% CI: 0.93–1.02) for the association between gestational diabetes and risk of macrosomia, 1.03 (95% CI: 0.98–1.08) for the association between maternal pregnancy smoking and risk of SGA, and 1.01–1.02 in the three comparison groups for the association between maternal pre-pregnancy BMI and risk of acute C-section.

**Table 3:** ORs and RORs with 95% CI on analyses A-E among the HOPE cohort and the source population

Associations	Crude OR				Adjusted^a^ OR				Adjusted^a^ ROR^b^	
	HOPE		Source		HOPE		Source			
	OR	95% CI	OR	95% CI	OR	95% CI	OR	95% CI	ROR	95% CI
Personal psychiatry – PPD										
Not having personal psychiatry	1.00	Reference	1.00	Reference	1.00	Reference	1.00	Reference	1.00	Reference
Having personal psychiatry	4.46	4.35–4.57	4.66	4.57–4.74	4.04	3.93–4.16	4.32	4.24–4.40	0.94	0.85–1.02
Family psychiatry – PPD										
Not having family psychiatry	1.00	Reference	1.00	Reference	1.00	Reference	1.00	Reference	1.00	Reference
Having family psychiatry	1.76	1.63–1.89	1.61	1.52–1.69	1.57	1.43–1.70	1.45	1.37–1.54	1.08	0.98–1.18
Gestational diabetes – macrosomia										
No gestational diabetes	1.00	Reference	1.00	Reference	1.00	Reference	1.00	Reference	1.00	Reference
Gestational diabetes	1.01	0.95–1.07	1.02	0.98–1.05	1.01	0.95–1.07	1.03	0.99–1.07	0.98	0.93–1.02
Smoking – SGA										
No smoking	1.00	Reference	1.00	Reference	1.00	Reference	1.00	Reference	1.00	Reference
Smoking	2.17	2.12–2.23	2.27	2.24–2.30	2.18	2.12–2.24	2.11	2.07–2.15	1.03	0.98–1.08
Maternal BMI – acute C-section										
12-18.4	1.00	Reference	1.00	Reference	1.00	Reference	1.00	Reference	1.00	Reference
18.5–24.9	1.23	1.14–1.31	1.21	1.16–1.26	1.17	1.08–1.26	1.16	1.11–1.21	1.01	0.94–1.08
25.0-29.9	1.73	1.64–1.82	1.71	1.66–1.77	1.68	1.59–1.77	1.66	1.61–1.71	1.01	0.94–1.08
30–50	2.20	2.11–2.29	2.18	2.12–2.23	2.18	2.09–2.28	2.13	2.08–2.19	1.02	0.95–1.10

^a^ adjusted for maternal age, parity, and education. ^b^ RORs were calculated as the ratio between adjusted OR in the HOPE cohort and the source population

*Abbreviations* Postpartum depression (PPD), babies born small for gestational age (SGA), Body Mass Index (BMI), cesarian section (C-section), odds ratio (OR), confidence interval (CI), relative odds ratio (ROR)

Sensitivity analyses restricted to singletons in analyses A and B similarly showed no difference between the two populations (Supplement Table S2). When restricting the analyses to Danish-born women, the HOPE cohort resembled the source population even more than without this restriction (Supplement Table S3).

## Discussion

The HOPE cohort encompasses information that spans the entire spectrum of PPD, with symptoms defined through the EPDS and diagnoses defined through hospital contacts and medication prescription fills. These data have been linked with comprehensive Danish register data, including detailed health and socioeconomic information and important PPD risk factors. The cohort comprises 170,218 childbirths, representing 38% of the Danish source population. Results suggest that cohort members are comparable to the source population, and moving forward, this cohort will provide new important knowledge for clinical practice, contributing to development of PPD prevention and treatment initiatives.

In the HOPE cohort, 7.8% exhibited PPD symptoms defined by EPDS screenings conducted within 12 weeks postpartum, while 3.1% had a PPD diagnosis defined through register information on medication prescriptions and hospital admissions within 6 months postpartum. The observed PPD prevalence of 3.1%, determined through registers, aligns with the prevalence in the source population (3.0%) and was expected to be lower than the estimated 10–15% prevalence in Western countries, as it reflects the treatment prevalence, and encompasses only more severe cases [1–3]. This is supported by findings from a study examining the agreement between self-report and register-based measurements of depression in the Danish population, which showed a significant underestimation of the true depression prevalence in the registers [19]. The 7.8% prevalence for PPD symptoms in the HOPE cohort aligns with previous findings in a report from the National Institute of Public Health in Denmark, showing that 7.5% of new mothers in Denmark have an EPDS score of 11 or above within the initial eight months postpartum [49]. When calculating the total PPD prevalence in the cohort, including both symptoms and diagnoses, we found a total prevalence of 10.1%, and observed an overlap of 0.8% between PPD defined through either symptoms or diagnosis. This very small overlap between symptoms and diagnosis clearly indicates that the HOPE cohort consists of a sample representing a wide spectrum of PPD, from mild to severe cases.

Comparison between the HOPE cohort and the source population showed that the main discrepancies between the two populations were related to an underrepresentation of women from foreign countries, consistent with research indicating that healthcare nurses are less inclined to screen women from foreign countries due to language barriers [50]. Moreover, a larger part of the source population than the cohort members had missing information on different characteristics. A sensitivity analysis demonstrated that missing numbers were largely due to a lack of information on women from foreign countries, as information before immigration to Denmark is not accessible in the Danish registers (Supplement Table S3). Additionally, the underrepresentation of women who gave birth to twins and triplets in the HOPE cohort may, in part, be attributed to the increased risk of preterm birth and subsequent hospitalization for those delivering twins and triplets [51]. Consequently, these individuals may not have been screened by the healthcare nurse at eight weeks postpartum.

Median EPDS scores over the years 2014–2021 were similar. The Covid-19 pandemic may explain part of the decline in the number of screenings in the years 2020 and 2021, as healthcare nurse visits were highly restricted during that time.

Examination of potential selection bias into the HOPE cohort produced reassuring results, with all associations demonstrating RORs containing 1 in the 95% CI, corresponding to no difference between the two populations.

### Strengths and limitations

It is a major strength to have data available on the combination of PPD symptoms and diagnoses for such a large cohort (170,218 childbirths) with the possibility of continuously update of register information. This cohort enables us to define a wider spectrum of PPD, making it possible to estimate prevalence and identify risk factors while taking the entire PPD spectrum into account.

Evaluation of potential selection bias was conducted on available information on five selected associations. However, we cannot rule out that selection bias may occur in other associations not investigated here. Thus, future studies should re-evaluate the representativity of the data source in relation to the specific study aims and details related to the applied study design. Nevertheless, the five exposure-outcome associations investigated were deliberately chosen, focusing on relevant perinatal associations to ensure reliability of the HOPE cohort within this research field, with reassuring results.

### Potential for future studies

The HOPE cohort was established with the aim of providing data to understand the causes and consequences of PPD, thereby creating a large knowledge base for clinical practice to support development of prevention and treatment strategies and initiatives. The cohort was also designed to be a source for making comparative studies on other mental disorders within the perinatal period and to examine psychotropic medication use during pregnancy and breastfeeding for mothers and offspring. Furthermore, PPD symptoms (EPDS) and diagnoses (register data on prescription medication and hospital contacts) on paternal PPD are available in the cohort, having this information on 45,738 parental dyads (mothers and fathers). The HOPE cohort has the potential to provide subtle and in-depth insights into knowledge of PPD.

## Electronic supplementary material

Below is the link to the electronic supplementary material.

Supplementary Material 1
