# Supplementary material for: The HOPE cohort: cohort profile and evaluation of selection bias
Source: Eur J Epidemiol. 2024 Aug 19;39(8):943–54. doi: 10.1007/s10654-024-01150-4 (PMC11410971; doi:10.1007/s10654-024-01150-4)
Supplement: Supplementary file 1 — Supplementary Material 1 [file 10654_2024_1150_MOESM1_ESM.pdf]

Supplemental content for the paper: "The HOPE cohort: cohort profile and evaluation of selection bias"

Table s1. Characteristics of the HOPE cohort and the source population on additional pregnancy- and birth-related complications with prevalence ratios and 95% confidence intervals.

Table s2. Analysis population A and B restricted to singletons.

Table s3. Characteristics of the HOPE cohort and the source population restricted to Danish-born women with prevalence ratios and 95% confidence intervals.

Table S1. Characteristics of the HOPE cohort and the source population on additional pregnancy- and birth-related complications with prevalence ratios and 95% confidence intervals.

	**HOPE cohort (n=170,218)**	**Source population (n=452,207)**	
**Variable**	**N (%)**	**N (%)**	**PR (95% CI)**
Preterm birth			
Yes	7,009 (4.1)	23,031 (5.1)	0.81 (0.80-0.81)
No	163,209 (95.9)	429,176 (94.9)	1.01 (1.01-1.01)
Postpartum haemorrhage			
Yes	13,035 (7.7)	31,623 (7.0)	1.10 (1.09-1.10)
No	157,183 (92.3)	420,584 (93.0)	0.99 (0.99-0.99)
Gestational hypertension			
Yes	4,301 (2.5)	9,491 (2.1)	1.20 (1.20-1.21)
No	165,917 (97.5)	442,716 (97.9)	1.00 (1.00-1.00)
Preeclampsia and/or eclampsia			
Yes	5,884 (3.5)	13,898 (3.1)	1.12 (1.12-1.13)
No	164,334 (96.5)	438,309 (96.9)	1.00 (1.00-1.00)
Previous abortion			
Yes	44,565 (26.2)	115,583 (25.6)	1.02 (1.02-1.03)
No	125,653 (73.8)	336,624 (74.4)	0.99 (0.99-0.99)
Hyperemesis gravidarum			
Yes	4,481 (2.6)	11,351 (2.5)	1.05 (1.05-1.05)
No	165,737 (97.4)	440,856 (97.5)	1.00 (1.00-1.00)

Abbreviations: Prevalence ratio (PR), confidence interval (CI)

Table s2. Analysis populations A and B restricted to singletons.

Associations	Crude OR				Adjusted[a] OR				Adjusted ROR[b]	
	HOPE		Source		HOPE		Source			
	OR	95% CI	OR	95% CI	OR	95% CI	OR	95% CI	ROR	95% CI
PH – PPD										
No PH	1.00	Reference	1.00	Reference	1.00	Reference	1.00	Reference	1.00	Reference
Having PH	4.48	4.36-4.59	4.54	4.47-4.62	4.06	3.94-4.17	4.27	4.19-4.34	0.95	0.86-1.04
FH – PPD										
No FH	1.00	Reference	1.00	Reference	1.00	Reference	1.00	Reference	1.00	Reference
Having FH	1.77	1.64-1.90	1.63	1.54-1.71	1.57	1.44-1.70	1.47	1.38-1.56	1.07	0.97-1.17

[a] adjusted for maternal age, parity, and education. [b] RORs were calculated as the ratio between adjusted OR in the HOPE cohort and the source population.

Abbreviations: Personal history of psychiatric disorders (PF), Postpartum depression (PPD), Family history of psychiatric disorders (FH), odds ratio (OR), confidence interval (CI), relative odds ratio (ROR)

Table S3. Characteristics of the HOPE cohort and the source population restricted to Danish-born women with prevalence ratios and 95% confidence intervals.

	HOPE cohort (n=147,235)	Source population (n=346,788)	
**Variable**	**N (%)**	**N (%)**	**PR (95% CI)**
PPD diagnosis			
Yes	4,799 (3.3)	11,583 (3.3)	0.98 (0.97-0.98)
No	142,436 (96.7)	335,205 (96.7)	1.00 (1.00-1.00)
Maternal age at delivery			
15-19 years	816 (0.6)	2,253 (0.6)	0.85 (0.85-0.86)
20-24 years	14,599 (9.9)	33,630 (9.7)	1.02 (1.02-1.03)
25-29 years	53,564 (36.4)	121,241 (35.0)	1.04 (1.04-1.04)
30-34 years	51,098 (34.7)	121,860 (35.1)	0.99 (0.98-0.99)
35-39 years	22,165 (15.1)	55,054 (15.9)	0.95 (0.94-0.95)
40-44 years	4,749 (3.2)	12,035 (3.5)	0.93 (0.93-0.93)
≥45 years	244 (0.2)	715 (0.2)	0.80 (0.80-0.81)
Calendar year of delivery			
2014	2,810 (1.9)	9.321 (2.7)	0.71 (0.71-0.71)
2015	18,272 (12.4)	46,190 (13.3)	0.93 (0.93-0.94)
2016	21,059 (14.3)	48,536 (14.0)	1.02 (1.02-1.03)
2017	22,311 (15.2)	47,839 (13.8)	1.10 (1.09-1.10)
2018	23,674 (16.1)	48,145 (13,9)	1.16 (1.15-1.16)
2019	23,670 (16.1)	48,151 (13.9)	1.16 (1.15-1.16)
2020	18,215 (12.4)	48,323 (13.9)	0.89 (0.88-0.89)
2021	17,224 (11.7)	50,283 (14.5)	0.81 (0.80-0.81)
Country of origin			
Denmark	147,235 (100.0)	346,788 (100.0)	
Foreign/unknown	0 (0.0)	0 (0.0)	
Cohabitation status			
Cohabitating	127,136 (86.3)	298,024 (85.9)	1.00 (1.00-1.01)
Not cohabitating	20,099 (13.7)	48,764 (14.1)	0.97 (0.97-0.97)
Education			
Mandatory	13,950 (9.5)	36,162 (10.4)	0.91 (0.90-0.91)
Short	45,693 (31.0)	106,440 (30.7)	1.01 (1.01-1.01)
Medium	7,657 (5.2)	17,796 (5.1)	1.01 (1.01-1.02)
High	79,795 (54.2)	185,742 (53.6)	1.01 (1.01-1.01)
Missing	140 (0.1)	648 (0.2)	0.51 (0.50-0.51)
Parity			
1	78,597 (53.4)	162,775 (46.9)	1.14 (1.13-1.14)
2	50,532 (34.3)	126,944 (36.6)	0.94 (0.93-0.94)
3	14,052 (9.5)	39,833 (11.5)	0.83 (0.83-0.83)
≥4	2,934 (2.0)	9,303 (2.7)	0.74 (0.74-0.75)
Missing	1,120 (0.8)	7,933 (2.3)	0.33 (0.33-0.34)
Singleton			
Yes	145,264 (98.7)	337,678 (97.4)	1.01 (1.01-1.01)
No	1,788 (1.2)	5,448 (1.6)	0.77 (0.77-0.78)
Missing	183 (0.1)	3,662 (1.1)	0.12 (0.11-0.12)
Obstetric Comorbidity Index			

0	98,760 (67.1)	232,824 (67.1)	1.00 (1.00-1.00)
1	31,330 (21.3)	72,072 (20.8)	1.02 (1.02-1.03)
2	10,223 (6.9)	24,323 (7.0)	0.99 (0.99-0.99)
≥3	6,922 (4.7)	17,569 (5.1)	0.93 (0.92-0.93)
Hospital depression diagnosis 1 year prior to delivery			
Yes	674 (0.5)	1,768 (0.5)	0.90 (0.89-0.90)
No	146,561 (99.5)	345,020 (99.5)	1.00 (1.00-1.00)
Antidepressant use 1 year prior to delivery			
Yes	5,298 (3.6)	12,965 (3.7)	0.96 (0.96-0.97)
No	141,937 (96.4)	333,823 (96.3)	1.00 (1.00-1.00)
Personal history of psychiatric disorders			
Yes	21,547 (14.6)	51,789 (14.9)	0.98 (0.98-0.98)
No	125,688 (85.4)	294,999 (85.1)	1.00 (1.00-1.00)
Family history of psychiatric disorders			
Yes	24,457 (16.6)	58,850 (17.0)	0.98 (0.98-0.98)
No	122,741 (83.4)	287,741 (83.0)	1.00 (1.00-1.01)
Missing	37 (0.0)	197 (0.1)	0.44 (0.44-0.45)
Gestational diabetes mellitus			
Yes	6,869 (4.7)	16,429 (4.7)	0.98 (0.98-0.99)
No	140,366 (95.3)	330,359 (95.3)	1.00 (1.00-1.00)
Macrosomia			
Yes	25,879 (17.6)	61,425 (17.7)	0.99 (0.99-1.00)
No	118,340 (80.4)	273,319 (78.8)	1.02 (1.02-1.02)
Missing	3,016 (2.0)	12,044 (3.5)	0.59 (0.59-0.59)
Smoking during pregnancy			
Yes	8,665 (5.9)	21,615 (6.2)	0.94 (0.94-0.95)
No	133,830 (90.9)	310,674 (89.6)	1.01 (1.01-1.02)
Missing	4,740 (3.2)	14,499 (4.2)	0.77 (0.77-0.77)
Small for gestational age			
Yes	13,575 (9.2)	30,744 (8.9)	1.04 (1.04-1.04)
No	130,521 (88.6)	303,707 (87.6)	1.01 (1.01-1.01)
Missing	3,139 (2.1)	12,337 (3.6)	0.60 (0.60-0.60)
Maternal pre-pregnancy BMI			
12-18.4	5,181 (3.5)	12,621 (3.6)	0.97 (0.96-0.97)
18.5-24.9	85,402 (58.0)	198,523 (57.2)	1.01 (1.01-1.02)
25-29.9	31,977 (21.7)	74,234 (21.4)	1.01 (1.01-1.02)
≥30-50	21,097 (14.3)	49,099 (14.2)	1.01 (1.01-1.02)
<12 or >50	1,221 (0.8)	3,454 (1.0)	0.83 (0.83-0.84)
Missing	2,357 (1.6)	8,857 (2.6)	0.63 (0.62-0.63)
Acute C-section			
Yes	20,869 (14.2)	50,324 (14.5)	0.98 (0.97-0.98)
No	126,366 (85.8)	296,464 (85.5)	1.00 (1.00-1.01)

Abbreviations: Postpartum depression (PPD), babies born small for gestational age (SGA), body mass index (BMI), caesarean section (C-section), prevalence ratio (PR), confidence interval (CI)
